# Development and validation of a self-management self-efficacy scale for premature birth prevention (SMSE-PBP) for women of childbearing age

**DOI:** 10.1186/s12905-024-02964-w

**Published:** 2024-02-20

**Authors:** Sun-Hee Kim, Yu-Jin Lee

**Affiliations:** College of Nursing, Research Institute of Nursing Science, Daegu Catholic University, 33, Duryugongwon-ro 17-gil, Nam-gu, Daegu, 42472 Republic of Korea

**Keywords:** Premature birth, Reproducibility of results, Self-efficacy, Self-management, Surveys, Questionnaires, Women of childbearing age

## Abstract

**Background:**

This study aimed to develop and evaluate the validity and reliability of a self-management self-efficacy for premature birth prevention (SMSE-PBP) in women of childbearing age (WCA).

**Methods:**

Instrument development and validation were undertaken in three phases: conceptualization, item generation and evaluation of content validity, and evaluation of construct and concurrent validity and reliability. Data were analyzed using exploratory and second-order confirmatory factor analyses, and concurrent validity was examined using Pearson’s correlation coefficients. The reliability was analyzed using omega hierarchical and Cronbach’s ⍺.

**Results:**

Content validity was assessed by experts and cognitive interviews of WCA. The SMSE-PBP consists of a second-order 3-dimension and 10-factor scale with 60 items; therefore, the construct and concurrent validity of the SMSE-PBP were supported. The omega values were 0.93 for pre-pregnancy SMSE-PBP, 0.92 for pregnancy SMSE-PBP, and 0.94 for hospital SMSE-PBP. Cronbach’s ⍺ was 0.88 for pre-pregnancy SMSE-PBP, 0.96 for pregnancy SMSE-PBP, and 0.96 for hospital SMSE-PBP.

**Conclusions:**

The SMSE-PBP scale is valid and reliable for WCA; it is helpful for WCA and health professionals to assess women’s SMSE-PBP and pre-pregnancy, pregnancy, or hospital SMSE-PBP. The next steps should include assessing the relationship with pregnancy health behaviors.

**Supplementary Information:**

The online version contains supplementary material available at 10.1186/s12905-024-02964-w.

## Background

Premature birth increases morbidity and mortality among children. Despite efforts to reduce preterm birth rates, premature births occur in an average of 10.6% (approximately 9–12%) of live births globally [1]. Additionally, South Korea had a total fertility rate of 0.81 in 2021; despite the ultra-low birth rate, the preterm birth rate was 9.2%, an increase of 1.5-fold compared with that in 2011 [2]. Therefore, preventive management to reduce the number of premature births is required.

Premature birth is caused by premature labor, preterm premature rupture of the membranes, and medically-intended preterm birth due to maternal and fetal complications [3]. Therefore, preventing premature labor and preterm premature rupture of the membranes and taking early action when symptoms occur can reduce the risk of premature birth. Women’s health before conception and during pregnancy is associated with preterm birth, and preventive measures can reduce the likelihood of preterm birth. For example, a woman’s body mass index before pregnancy as well as smoking, birth spacing, unintended pregnancies, gestational age [4, 5], and preconception care [6] are modifiable risks factors for preterm birth. Additionally, maternal diet quality [7, 8] and healthy dietary patterns [9, 10] during pregnancy are associated with a lower risk of preterm birth. Managing such chronic conditions before and during pregnancy helps prevent premature birth [3].

Self-management behaviors through information, support, and collaboration can be adopted [11]. Self-efficacy is the perceived ability to follow the necessary health behaviors, despite various situations or obstacles [12], and is essential for self-management [13]. Therefore, if women of childbearing age (WCA) at risk of preterm birth recognize self-efficacy in self-management for preterm birth prevention, they can be motivated to self-reinforce their lack of behavioral competence.

However, it is difficult to evaluate the level of self-management self-efficacy for premature birth prevention (SMSE-PBP) because there is no appropriate measurement scale currently. The Self-Rated Abilities for Health Practices: health self-efficacy (SRAHP) self-management measurement scale was previously developed [14]. However, because the SRAHP assesses self-management for general health, it does not address preterm birth prevention before or during pregnancy.

Therefore, this study aimed to develop an SMSE-PBP scale using Blok’s conceptual definition of self-management behavior [11] as a basic framework to obtain a scale that might help WCA controlling premature birth risk factors before and during pregnancy. Our findings will help community health care centers and hospitals evaluate the SMSE-PBP in WCA and develop effective interventions.

## Methods

### Study design

This study aimed to develop and evaluate an SMSE-PBP for WCA. We used the diagnostic accuracy studies: Standards for the Reporting of Diagnostic Accuracy Studies, as guidance for writing this article.

### Conceptualization

#### Theoretical framework and literature review

We performed a literature review based on the attributes of self-management behaviors. This concept includes seven attributes classified as proactive, reactive actions, and dynamic process [11]. In this study, we attempted to integrate self-efficacy with the perception of one’s ability to engage in self-management behaviors.

We searched the literature in the PubMed, Embase, Cochrane Library, and CINAHL databases. Articles written in English or Korean with full text were included. The search was performed by combining controlled and text words. Finally, 32 articles were reviewed. The data extracted were organized by attributes of self-management behavior [11].

In the literature review, we confirmed that premature birth prevention had three dimensions, pre-pregnancy period, during pregnancy, and after hospital admission and discharge due to risk symptoms; and that the dimensions of SMSE-PBP were sequentially moved from one dimension to another dimension because the premature birth risk changes over time. In addition, this study found that important attributes of self-management changed according to these dimensions as the pregnancy progressed and as the environment in which the women were placed changed. Therefore, as the attributes in these dimensions are highly correlated over time, the dimensions can only be explained by higher-order factors when several dimensions are evaluated simultaneously.

#### Qualitative interviews

One-on-one in-depth interviews were conducted with women who had experienced premature birth or those not at risk of preterm birth. There were 34 interviewees who were married and aged between 29 and 47 years. The number of premature births ranged from 0 to 2.

The self-management behavior concept attributes, literature reviews, and content analysis results of the interviews were integrated. Consequently, the SMSE-PBP includes three dimensions: pre-pregnancy, during pregnancy, and after admission and discharge.

As a result, SMSE-PBP was defined by the following dimensions: WCA have a proactive lifestyle that includes “diet and adequate nutrition,” “stress reduction,” “exercises/posture, adequate sleep/rest,” and “generally healthy lifestyle, hygiene, and body function promotion;” proactive problem specific management that includes “trigger management, control of fatigue, and lifestyle adherence” and “treatment adherence, oversight of care, safety management;” and proactive collaboration that includes “check-ups” and “communication with primary care provider (PCP)” in the pre-pregnancy period to prevent premature birth. During pregnancy to prevent premature birth, WCA have a proactive lifestyle that includes “stress reduction,” “exercises/posture and adequate sleep/rest” and “general healthy lifestyle, hygiene, and body function promotion;” proactive problem-specific management that includes “trigger management, control of fatigue, and lifestyle adherence,” “treatment adherence, oversight of care, and safety management,” and “self-monitoring;” reactive management that includes “distancing from triggers,” “hospital visit and help-seeking,” “knowing when to get help, management of complications, coping in a high-risk situation, and adaptation,” and “observe/tracking symptoms;” and proactive collaboration that includes “check-ups,” “communication with PCP,” “following plan of care from PCP,” and “family support and feedback seeking”.

Finally, WCA demonstrate reactive management that includes “distancing from trigger” and “observing/tracking symptoms,” proactive collaboration that includes “cooperative treatment with PCP” and “family support and feedback seeking,” proactive mental support that includes “persistence and information management” and “healthy coping,” and proactive planning that includes “goal setting” after hospital admission and discharge to prevent premature birth.

### Item generation and evaluation of content validity

#### Item generation

The initial items were developed based on three dimensions. The three dimensions were named pre-pregnancy SMSE-PBP (dimension 1) for the pre-pregnancy preparation period, pregnancy SMSE-PBP (dimension 2) for during pregnancy and before hospital admission, and hospital SMSE-PBP (dimension 3) for after hospital admission discharge. A total of 15, 55, and 22 initial items were developed for dimensions 1, 2, and 3, respectively.

Responses to the questions were measured using a 5-point Likert scale ranging from “I can hardly do it” (1 point) to “I can do it very well” (5 points).

#### Expert content validity assessment

The content validity tests were conducted in triplicate. The experts were all female aged 41 to 53 years, with an average clinical experience of 12.3 years. For the third test, six experts gathered online for 2 h to review the entire set of items, sub-categories, and dimensions, and they discussed and proposed modifications and deletions of items with an unacceptable item content validity index (I-CVI) in the second test. After the cognitive interviewing, four experts convened in an online meeting to modify words that women found difficult to understand into words that were easy to understand.

The content validity was evaluated in terms of relevance and comprehensiveness. The suitability of the sub-categories and items ranged from “not relevant” (1 point) to “very relevant” (4 points), and the comprehensiveness of the sub-categories’ items ranged from “very uncomprehensive” (1 point) to “very comprehensive” (4 points), both measured using a 4-point Likert scale. The I-CVI of the SMSE-PBP scale was set as the criterion for validity with a score of ≥ 0.78 [15]. For scale-level content validity, the average of all items’ I-CVI (S-CVI/Ave) was set as the criterion for validity with a score of ≥ 0.90 [15], and the ratio of the items that all experts judged as appropriate (S-CVI/UA) was set at 0.70 as the criterion [15].

#### Cognitive interviewing

After three reviews of content validity, the researchers met one-on-one with WCA through face-to-face, telephone, or online conversations to assess their understanding (comprehensibility) of the revised items on the SMSE-PBP scale using the cognitive interviewing method, receiving feedback for modifications [16]. A total of 10 WCA participated in cognitive interviews; ages ranged from 19 to 43 years, including 2 in their teens, 2 in their 20s, 3 in their 30s, and 3 in their 40s.

### Evaluation of construct validity and reliability

#### Study participants

The target population for the validity and reliability assessment of the survey consisted of WCA residing in South Korea, aged 19 to 49, who expressed an intention for future childbirth. A total of 795 data points were collected. Among them, 97 respondents who did not complete the survey (*n* = 91) and those who responded with only 1 number (*n* = 6) were excluded, resulting in a final sample of 698. Data were randomly allocated to the two groups by block randomization using a random sequence of four block sizes in Excel. Ultimately, 349 data points were used for the exploratory and confirmatory factor analyses.

To determine the sample size for the initial model, a CFA model with 84 items, we followed the method proposed by Tak [17]. According to this method, a sample size can be considered adequate when the number of cases is 200 or more, or when the ratio of cases to measured variables is 5 to 1 or higher [17]. According to the other method proposed by MacCallum et al. [18], when Cronbach’s alpha set at 0.05, degrees of freedom at 3386, Power at 0.90, null the root-mean-square error of approximation (RMSEA) at 0.00, and alternative RMSEA at 0.05, the minimum sample size was estimated to be 31. And sample size considering the dropout rate (20%) for online surveys was 250. Therefore, the sample size met the minimum criteria for statistical testing.

### Ethical considerations

This study was approved by the institutional review board of the researcher’s affiliated university (approval no: CUIRB-2021-0076-02) before the start of the survey. The entire research process was conducted in accordance with the committee’s ethical guidelines and regulations. The questionnaire included the SMSE-PBP scale, Korean (K)-SRAHP, and demographic characteristics. All survey participants read the online research description, voluntarily participated, agreed to provide informed consent, and received remuneration (approximately USD 3.5) for completing the questionnaire.

### Data collection

Recruitment documents were posted on mobile messenger and social network services (Twitter and Facebook) to recruit participants from March 20, 2023 to April 3, 2023. A total of 128 responses were recorded during this period. Subsequently, a research agency was commissioned to conduct the survey, considering the population distribution and recruiting participants from different regions nationwide. The company conducted an online survey from April 5, 2023 to April 10, 2023 and collected additional 576 responses.

### Data analysis

To confirm the construct validity of the SMSE-PBP, a factor analysis was conducted, followed by concurrent validity and reliability analyses. The specific data analysis method was as follows: IBM SPSS Statistics for Windows (version 25.0; Armonk, NY, USA) was used for the exploratory factor analysis (EFA), Pearson’s correlation, Cronbach’s ⍺, and demographic characteristic analysis. The LAVAAN package in R was used for confirmatory factor analysis (CFA) and reliability analyses (coefficient omega). When conducting an online survey, the items of the measurement tool were set as mandatory responses, ensuring no missing data occurred.

#### Construct validity

The theory of self-management behavior has not been sufficiently applied since its announcement in 2017 [11]. Cross-validation was performed for construct validity because, compared with non-pregnant women, pregnant women have different motivating factors for self-management [19].

Step 1: To perform EFA, a principal axis factor analysis was conducted using promax rotation owing to the correlation between the factors. The Kaiser (Mayer) Olkin test and Bartlett’s test of sphericity were used to assess the appropriateness of the data for factor analysis. The item selection criteria for factor extraction had a correlation coefficient < 0.80 distribution with a factor loading value > 1, commonality greater than 0.30, regression coefficient > 0.40 in the pattern matrix, and correlation coefficient < 0.50 in several factors at the same time in the structure matrix. One factor comprised at least three items [20].

Step 2: CFA was conducted for each dimension; owing to the high correlation between dimensions, a higher-order CFA was conducted. The model fit was evaluated according to the acceptability cutoff value of each fit index. The model fit indices were the normed χ^2^ (NC < 5), comparative fit index (CFI ≥ 0.90), the Tucker–Lewis index (TLI ≥ 0.90) [21], RMSEA (< 0.08) [22], standardized root-mean-square residual (SRMR < 0.08) [23], and Akaike information criterion (AIC; the lowest). In addition, the standardized factor loadings, R square, and variance, which are model estimates, were checked. The items were deleted after considering the modification indices (MI), standardized factor loadings, error variance, and importance of the items.

Step 3: The convergence validity of the component factors by each dimension was confirmed by standardized factor loadings ≥ 0.50 (*P* < 0.05), construct reliability (CR) ≥ 0.70, and average variance extracted (AVE) ≥ 0.50. The discriminant validity of the component factors for each dimension was confirmed by the fact that the correlation coefficient between the factors was < 0.80, and the AVE of the latent variables was greater than the squared value of the correlation coefficient between the latent variables (AVE > Φ^2^) [24].

#### Concurrent validity

In this study, Pearson’s correlation coefficient with the K-SRAHP [25] was used for concurrent validity analysis. The SRAHP was developed by Becker et al. [14] and translated into Korean and evaluated for validity and reliability by Lee et al. [25]. It is widely used in South Korea. The scale comprises 23 items with 4 sub-categories. Each item is measured on a 5-point Likert scale ranging from strongly disagree (1 point) to strongly agree (5 points), and the total score ranges from 24 to 120. Higher scores indicate higher health self-efficacy. The Cronbach’s ⍺ was 0.91 in Lee et al. [25] and 0.94 in this study.

#### Reliability analysis

To confirm the reliability, Cronbach’s ⍺, 95% confidence intervals (95% CI), and the coefficient omega (ω) of each dimension were calculated. The hierarchical omega (ω_h_) of a second-order factor in all dimensions was calculated. The significance level for all statistical data was set at *P* < 0.05.

## Results

### Evaluation of content validity

In the first content validity test, based on the advice of the experts, the sentences were revised to increase clarity and readability, and four items were added. Finally, there were 16, 57, and 23 items in dimensions 1, 2, and 3, respectively.

In the second content validity test, dimension 1 had no items with an I-CVI < 0.78, and the phrases were revised. The scale-level CVI for S-CVI/Ave and S-CVI/UA was 1.00. In dimension 2, there were 10 items with an I-CVI < 0.78; however, the phrases were not deleted, and S-CVI/Ave and S-CVI/UA were 0.94 and 0.79, respectively. In dimension 3, no items had an I-CVI < 0.78. The S-CVI/Ave and S-CVI/UA were 1.00. After the second content validity test, the sentences were revised, one item was deleted, and two items were merged into one through expert consensus meetings. Subsequently, upon expert review, 10 items with I-CVI < 0.78 in dimension 2 were deleted. Consequently, there were finally 16, 45, and 23 items in dimensions 1, 2, and 3, respectively.

After cognitive interviewing, complicated terms and phrases, such as folic acid, rubella, autoimmune disease, advanced medical institution, and contraction of the lower abdomen, were modified into understandable expressions. The final preliminary items consisted of 6, 6, and 4 items in the 3 sub-categories of dimension 1 (the proactive lifestyle before pregnancy, the proactive problem-specific management before pregnancy, and the proactive collaboration before pregnancy, respectively); 18, 10, 5, 6, and 6 items in the 5 sub-categories of dimension 2 (proactive lifestyle during pregnancy, proactive collaboration during pregnancy, reactive management of risk symptom recognition during pregnancy, reactive management according to risk symptoms during pregnancy, and self-monitoring of risk symptoms during pregnancy, respectively); and 6, 3, 3, 5, and 6 items in the 4 sub-categories of dimension 3 (management of triggers after hospital admission, tracking management of symptoms after hospital admission, proactive collaboration after hospital admission, proactive support after hospital admission, and reactive management of the disease after discharge, respectively).

### Evaluation of construct and concurrent validity and reliability

#### General characteristics of participants

The mean (standard deviation [SD]) age of the 698 participants was 31.88 (5.93) years; 542 (77.7%) participants had an associate or bachelor’s degree, 564 (80.8%) were employed, 415 (59.5%) were married, 406 (58.2%) had insufficient monthly income, and 386 (55.3%) lived in metropolitan cities. Most participants (75.8%) had no childbirth, the mean (SD) number of childbirths was 0.35 (0.67), and the mean (SD) age at first childbirth was 30.82 (3.98) years. Most participants (88.2%) reported no health issues immediately after childbirth, 18 reported that their first baby was sick immediately after birth, and 20 received perinatal hospital care for a disease. The mean (SD) number of premature births was 1.21 (0.47), and 313 (44.8%) participants were perceived to be at moderate risk of premature birth, whereas 430 (61.6%) received premature birth education (Table 1). The demographic characteristics of the participants who were included in the exploratory and confirmatory factor analyses are presented in Table 1.

**Table 1:** Demographic characteristics of participants

Variables	Total (*N* = 698)	Explore factor analysis (*n* = 349)	Confirmatory factor analysis (*n* = 349)
**Age (year), mean (SD)**	31.88 (5.93)	31.97 (5.96)	31.78 (5.89)
**Education, n (%)**			
High school or below	74 (10.6)	37 (10.6)	37 (10.6)
Associate or Bachelor’s degree	542 (77.7)	270 (77.4)	271 (77.7)
Master’s degree or higher	82 (11.7)	42 (12.0)	41 (11.7)
**Employment, n (%)**			
Yes	564 (80.8)	280 (80.2)	284 (81.4)
No	134 (19.2)	69 (19.8)	65 (18.6)
**Marriage status, n (%)**			
Single	279 (40.0)	138 (39.5)	141 (40.4)
Married	415 (59.5)	208 (59.6)	207 (59.3)
Divorced or bereaved	4 (0.5)	3 (0.9)	1 (0.3)
**Monthly income, n (%)**			
Very insufficient	54 (7.7)	34 (9.7)	20 (5.7)
Insufficient	406 (58.2)	199 (57.0)	207 (59.3)
Sufficient	234 (33.5)	113 (32.4)	121 (34.7)
Very sufficient	4 (0.6)	3 (0.9)	1 (0.3)
**Residential area, n (%)**			
Metropolitan city	386 (55.3)	188 (53.9)	198 (56.7)
Province	312 (44.7)	161 (46.1)	151 (43.3)
**Childbirth**			
Yes	169 (24.2)	87 (24.9)	82 (23.5)
No	529 (75.8)	262 (75.1)	267 (76.5)
**Number of childbirths**^a^, **mean (SD)**	0.35 (0.67)	0.33 (0.62)	0.36 (0.71)
**Age at first childbirth**^a^, **mean (SD)**	30.82 (3.98)	30.92 (3.79)	30.71 (4.19)
**Health issues of the baby immediately after childbirth**^a^, **n(%)**			
Yes	20 (11.8)	12 (13.6)	8 (9.8)
No	150 (88.2)	76 (86.4)	74 (90.2)
**Baby who was sick right after birth**^**b**^ **(Duplicate selection), n (%)**			
First	18 (85.7)	10 (83.3)	8 (88.9)
Second	3 (14.3)	2 (16.7)	1 (11.1)
**Perinatal hospital care for a disease**^**b**^, **n (%)**			
Yes	20 (18.0)	11 (20.0)	9 (16.1)
No	91 (82.0)	44 (80.0)	47 (83.9)
**Number of premature births**^**b**^, **mean (SD)**	1.21 (0.47)	1.24 (0.50)	1.19 (0.43)
**Risk of premature birth, n (%)**			
Very low	62 (8.9)	31 (8.9)	31 (8.9)
Low	188 (26.9)	93 (26.6)	95 (27.2)
Moderate	313 (44.8)	159 (45.6)	154 (44.1)
High	120 (17.2)	57 (16.3)	63 (18.1)
Very high	15 (2.2)	9 (2.6)	6 (1.7)
**Premature birth education, n (%)**			
Yes	430 (61.6)	216 (61.9)	214 (61.3)
No	268 (38.4)	133 (38.1)	135 (38.7)

^a^Answered by those who had childbirth experience; ^b^Missing data was excluded. *Abbreviations* SD, standard deviation

### Construction validity

#### Step 1: EFA

After multiple rounds of EFA, the items were systematically removed one by one in each successive EFA for each dimension. Dimension 1 resulted in a 3-factor scale with 13 items. Dimension 2 resulted in a 5-factor scale with 32 items. Dimension 3 resulted in a 2-factor scale with 16 items.

#### Step 2: CFA

The CFA for each dimension and the second-order model were implemented while comparing the model fit indices. For each CFA, model modification or item deletion was considered while reviewing MI, model estimates, and the interpretability and importance of the items.

The first CFA for dimension 1 was conducted to test a 3-factor scale with 13 items. During the fourth CFA, three covariances were set sequentially between the two items. The final model fit statistics presented in Table 2 were acceptable. The standardized factor loadings ranged 0.55–0.82, except 0.31 for item 1. R squares ranged 0.01–0.67, and the standardized variances ranged 0.33–0.91 (Appendix S1).

**Table 2:** Fit indices of the first- and second-order confirmatory factor models of the SMSE-PBP (*N* = 349)

Model	χ^2^	df	P value	NC	CFI	TLI	RMSEA (90% CI)	SRMR	AIC
Dimension 1: the first	274.36	62	< 0.001	4.23	0.90	0.88	0.10 (0.09–0.11)	0.06	9704.07
Dimension 1: the final	158.15	59	< 0.001	2.68	0.96	0.94	0.07 (0.06–0.08)	0.05	9593.85
Dimension 2: the first	1359.48	454	< 0.001	3.07	0.88	0.87	0.08 (0.07–0.08)	0.07	22911.21
Dimension 2: the final	1118.79	449	< 0.001	2.49	0.92	0.91	0.07 (0.06–0.07)	0.06	22644.52
Dimension 3: the first	466.90	103	< 0.001	4.53	0.93	0.92	0.101 (0.09–0.11)	0.04	9313.41
Dimension 3: the final	265.95	86	< 0.001	3.09	0.96	0.95	0.08 (0.07–0.09)	0.03	8601.78
First order SMSE-PBP	3449.31	1654	< 0.001	2.09	0.89	0.89	0.06 (0.05–0.06)	0.06	39844.86
Second-order SMSE-PBP: the first	3676.82	1686	< 0.001	2.18	0.88	0.88	0.06 (0.06–0.06)	0.07	40008.36
Second-order SMSE-PBP: the final	3492.11	1684	< 0.001	2.07	0.89	0.89	0.06 (0.05-0.06)	0.06	39827.65

**Abbreviations** SMSE-PBP, Self-Management Self-Efficacy Scale for Premature Birth Prevention; df, degree-of-freedom; NC, normed chi-square; CFI, Comparative Fit Index; TLI, Tucker-Lewis Index; RMSEA, Root Mean Square Error of Approximation; SRMR, Standardized Root Mean Residual; AIC, Akaike Information Criterion

Dimension 1, Pre-pregnancy SMSE-PBP; Dimension 2, Pregnancy SMSE-PBP; Dimension 3, Hospital SMSE-PBP

Fit indices: NC (< 3 good, < 5 acceptable), CFI, TLI (> 0.9), RMSEA, SRMR (< 0.08), AIC (the lowest)

The first CFA for dimension 2 was conducted to test a 5-factor scale with 32 items. During the sixth CFA, five covariances were set sequentially between the two items. The final model fit statistics presented in Table 2 were acceptable. The standardized factor loadings ranged 0.56–0.85. R squares ranged 0.31–0.73, and the standardized variances ranged 0.27–0.69 (Appendix S1).

The first CFA for dimension 3 was conducted to test a 2-factor scale with 16 items. During the fourth CFA, three covariances were set sequentially between the two items. The MI for the covariance between items 15 (seeking support from family or acquaintances) and 22 (seeking support from family or acquaintances after discharge) was 39.79, and the content of the 2 sentences was similar; therefore, item 22 was deleted. The final model fit statistics presented in Table 2 were acceptable. The standardized factor loadings ranged 0.71–0.89. R squares ranged 0.51–0.79, and the standardized variances ranged 0.21–0.49 (Appendix S1).

**Fig. 1:**
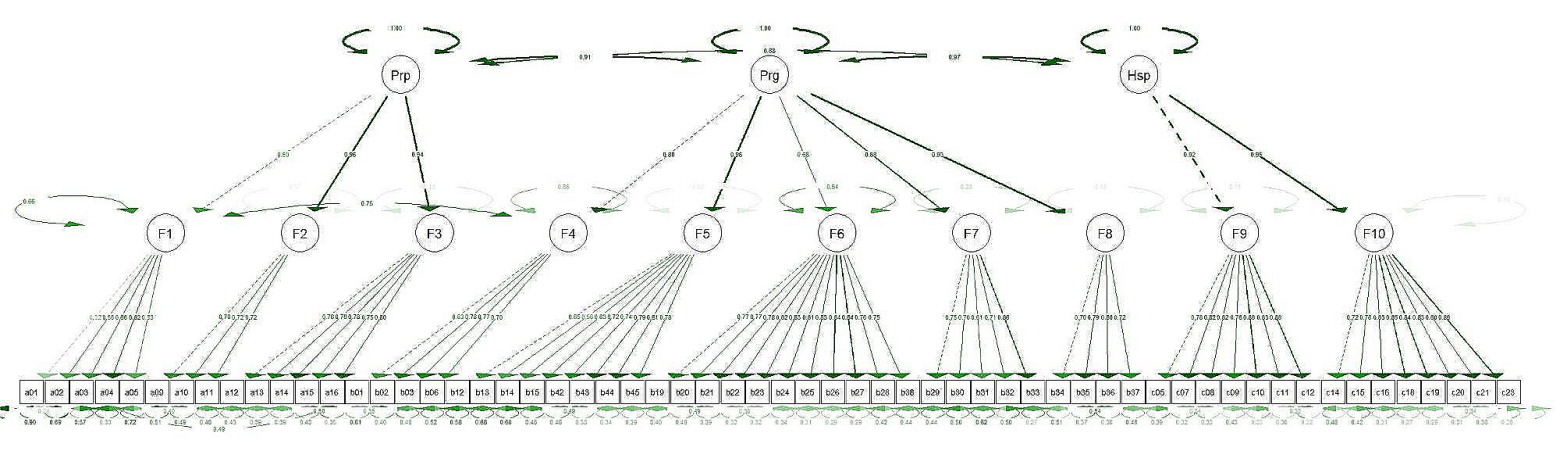
Second-order confirmation factor model of the SMSE-PBP including standardized estimations. *Abbreviations:* F1, proactive lifestyle before pregnancy; F2, proactive problem-specific management before pregnancy; F3, proactive collaboration before pregnancy; F4, proactive lifestyle during pregnancy; F5, proactive collaboration during pregnancy; F6, reactive management of risk symptom recognition during pregnancy; F7, reactive management according to risk symptoms during pregnancy; F8, self-monitoring of risk symptoms during pregnancy; F9, proactive collaboration and tracking management of symptoms after hospital admission; F10, proactive support and reactive management of the disease after discharge; Prp, Pre-pregnancy SMSE-PBP; Prg, Pregnancy SMSE-PBP; Hsp, Hospital SMSE-PBP

Next, the first CFA for the three dimensions was conducted to test a 10-factor scale for the first-order SMSE-PBP model, and a second-order CFA was conducted using the three dimensions as latent variables. During the second CFA, two covariances were set sequentially between two latent variables and two items (Fig. 1). The final second-order model fit statistics presented in Table 2 were acceptable.

#### Step 3: convergent and discriminant validity

In the second-order factor model, the standardized factor loadings ranged 0.53–0.88 (criteria > 0.5), except 0.32 for item 1 of pre-pregnancy SMSE-PBP for observed variables and 0.59–0.96 for latent variables of the first order. R squares, the standardized variances, the CR, and the AVE were acceptable, supporting the convergent validity of the component factors (Appendix S1).

In the first step of the second-order factor analysis, the correlation coefficient between 10 factors ranged 0.16–0.40, which was acceptable and AVE > Φ^2^. Therefore, the discriminant validity of the component factors was supported (Appendix S1).

#### Concurrent validity

The Pearson’s correlation coefficient between the SMSE-PBP included all dimensions and the K-SRAHP was 0.73 (*P* < 0.001); K-SRAHP was 0.61 (*P* < 0.001) between dimension 1, 0.69 (*P* < 0.001) between dimension 2, and 0.68 (*P* < .001) between dimension 3.

#### Reliability

The Cronbach’s ⍺ (95% CI) was 0.88 (0.87–0.89) for dimension 1, 0.96 (0.95–0.96) for dimension 2, and 0.96 (0.95–0.96) for dimension 3. The Cronbach’s ⍺ (95% CI) for factors ranged from 0.76 (0.73–0.79) to 0.96 (0.95–0.96) (Table 3). ω_*h*_ was 0.93 for dimension 1, 0.92 for dimension 2, and 0.94 for dimension 3. ω for factors ranged from 0.70 to 0.95 (Table 3).

**Table 3:** Reliability of the SMSE-PBP (*N* = 349)

Dimensions/Factors	Cronbach’s α (95% CI)	ω or ω_h_
**Pre-pregnancy SMSE**		
Sub-total	0.88 (0.87–0.89)	.93^a^
Factor 1	0.76 (0.73–0.79)	0.70
Factor 2	0.79 (0.76–0.82)	0.70
Factor 3	0.87 (0.85–0.88)	0.86
**Pregnancy SMSE**		
Sub-total	0.96 (0.95–0.96)	.92^a^
Factor 4	0.82 (0.79–0.84)	0.77
Factor 5	0.89 (0.87–0.90)	0.88
Factor 6	0.95 (0.94–0.95)	0.95
Factor 7	0.85 (0.83–0.87)	0.85
Factor 8	0.85 (0.83–0.86)	0.81
**Hospital SMSE**		
Sub-total	0.96 (0.95–0.96)	.94^a^
Factor 9	0.93 (0.93–0.94)	0.93
Factor 10	0.93 (0.92–0.94)	0.93

**Abbreviations** CI, confidence interval; SMSE-PBP, Self-Management Self-Efficacy Scale for Premature Birth Prevention

F1, proactive lifestyle before pregnancy; F2, proactive problem-specific management before pregnancy; F3, proactive collaboration before pregnancy; F4, proactive lifestyle during pregnancy; F5, proactive collaboration during pregnancy; F6, reactive management of risk symptom recognition during pregnancy; F7, reactive management according to risk symptoms during pregnancy; F8, self-monitoring of risk symptoms during pregnancy; F9, proactive collaboration and tracking management of symptoms after hospital admission; F10, proactive support and reactive management of the disease after discharge; ω, coefficient omega; ^a^ω_*h*_, hierarchical omega

#### Final SMSE-PBP scale

The final scale consisted of a second-order 3-dimension and a 10-factor scale with 60 items. The pre-pregnancy SMSE-PBP (dimension 1) consisted of a 3-factor scale with 13 items. The pregnancy SMSE-PBP (dimension 2) consisted of a 5-factor scale with 32 items. The hospital SMSE-PBP (dimension 3) consisted of a 2-factor scale with 15 items (Appendix S1, S2).

## Discussion

The items of the SMSE-PBP were developed based on the attributes of self-management behavior [11]. Five of six attributes of self-management behaviors, excluding dynamic process, were identified as factors in this study. This scale was developed to test the concept of self-management behaviors [11], which was well supported in this study. While developing the SMSE-PBP scale, content validity was tested three times with experts. Following previous recommendations [26, 27], the relevance, comprehensiveness, and comprehensibility of construct concepts were evaluated in this study.

Furthermore, construct validity was confirmed for each of the three dimensions and the second-order factor model in this study. The model fit indices of each dimension reached acceptable levels [22, 23], and the convergent and discriminant validity were appropriate. In this study, the CRs and AVEs of all items, except for item 1 for the pre-pregnancy SMSE-PBP dimension, were high. However, item 1 concerned adjusting to an average weight before pregnancy, and although the regression coefficient was low at 0.32, it was not deleted as it was considered essential since obesity and underweight influence preterm birth [28–31]. On the other hand, a scale with discriminant validity should have a factor correlation below 0.80 [32], which was observed for all factor correlations in this study. Therefore, the SMSE-PBP of the three dimensions and the second-order factor model can be used for the independent assessment of WCA.

In the second-order factor model, the covariances between latent variable 1 of dimension 1 and latent variable 4 of dimension 2 were set, as they regard factors related to a proactive lifestyle before and during pregnancy. Furthermore, items on nutritional intake, stress management, and health management in daily life were included. Therefore, the concurrent validity of the SMSE-PBP was confirmed, and each dimension and the K-SRAHP showed a significant correlation (range of correlation coefficient = 0.61–0.69). The SMSE-PBP, pre-pregnancy SMSE-PBP, pregnancy SMSE-PBP, and hospital SMSE-PBP are measurements that can accurately evaluate the attributes of SMSE.

In this study, ω_*h*_ reliability was calculated because it is useful and highly advantageous for scales that may be multidimensional [33]. . Although we presented ω_*h*_ and Cronbach’s ⍺ in this study, ω_*h*_ estimates have been reported to be unbiased and more accurate than Cronbach’s ⍺. Considering that ω for all items of the second-order model and all sub-categories was above 0.69, it exceeded the recommended ≥ 0.70 level [34].

The primary objective of this tool is to serve as a screening instrument, guiding clinical interventions for the prevention of preterm birth. Applied effectively, this tool has the potential to identify modifiable risk factors in women, allowing for the clear distinction of these risks. Subsequently, it can play a pivotal role in guiding early healthcare interventions to effectively mitigate such identified risks.

In the context of this study, the tool was applied to WCA. However, if considering the application of this tool to pregnant women, it is imperative to confirm its applicability through a thorough re-evaluation of validity before actual use. This cautious approach ensures that the tool’s effectiveness and relevance are validated in the specific context of pregnant women.

Moreover, through the identification of modifiable risk factors, the tool may contribute to facilitating the interaction between women’s factors of SMSE-PBP and healthcare interventions, thereby potentially preventing adverse birth outcomes. This multifaceted approach underscores the significance of the tool in addressing various aspects related to preterm birth prevention.

## Strengths and limitations

We developed the SMSE-PBP with validity and reliability because it was difficult to find a self-reported scale to evaluate the self-efficacy of self-management for premature birth prevention in WCA. Our scale includes a wide range of factors and can be used to evaluate preventive self-health management according to the trajectory of disease occurrence, exacerbation, and recurrence.

While the scale demonstrates notable strengths, it is essential to acknowledge its limitations. Data collection, while comprehensive across South Korea, lacked random sampling and involved a relatively small sample size, impacting the generalizability and interpretation of results. For future studies, the validation process of this scale should be extended to a larger and more diverse random sample. Investigating the applicability of each dimension scale across different populations, such as pre-pregnancy, pregnant, and hospitalized pregnant women, is crucial. Moreover, its predictive validity of whether women with high SMSE-PBP a low incidence of premature birth have should also be assessed. It is also imperative to estimate response times for the full set of 60 items and reassess the scale’s potential as a screening tool in public healthcare systems. Additionally, test-retest reliability should be evaluated in a future study since it was not evaluated here.

Caution is advised in the application, particularly for individuals experiencing heightened stress or anxiety related to pregnancy. This is crucial as there is a possibility that stress, or anxiety may inadvertently increase. To gain a better understanding of potential correlations, future studies should assess the scale’s application and its relationship with stress or anxiety related to pregnancy.

## Conclusions

In conclusion, we developed a SMSE-PBP scale for WCA and evaluated the content, construct, concurrent validity, and internal consistency reliability of the scale. In future studies, the predictive validity and measurement invariance and stability reliability of the scale must be evaluated. This scale can be applied in a self-reported form to WCA.

### Electronic supplementary material

Below is the link to the electronic supplementary material.

**Appendix S1.** Convergent and discriminant validity of the SMSE-PBP (N=349).

**Appendix S2.** Self-Management Self-Efficacy Scale for Premature Birth Prevention (English Version).

## Data Availability

Datasets used/or analyzed during the current study are available from the corresponding author on reasonable request.

## Abbreviations

AIC: Akaike information criterion
AVE: average variance extracted
CFA: confirmatory factor analysis
CFI: comparative fit index
CR: construct reliability
EFA: exploratory factor analysis
I-CVI: item content validity index
K-SRAHP: Korean Self-Rated Abilities for Health Practices
MI: modification indices
NC: normed chi-square
PCP: primary care provider
RMSEA: root-mean-square error of approximation
S-CVI/Ave: average of all items’ I-CVI
S-CVI/UA: ratio of the items judged as appropriate by all experts
SMSE-PBP: self-management self-efficacy scale for premature birth prevention
SRAHP: Self-Rated Abilities for Health Practices:health self-efficacy
SRMR: standardized root-mean-square residual
TLI: Tucker–Lewis index
WCA: women of childbearing age
