# Supplementary material for: Development and validation of a self-management self-efficacy scale for premature birth prevention (SMSE-PBP) for women of childbearing age
Source: BMC Womens Health. 2024 Feb 20;24:134. doi: 10.1186/s12905-024-02964-w (PMC10877791; doi:10.1186/s12905-024-02964-w)
Supplement: Supplementary file 1 — Appendix S1. Convergent and discriminant validity of the SMSE-PBP (N=349). [file 12905_2024_2964_MOESM1_ESM.docx]

**Additional file 1**

**Appendix S1. Convergent and discriminant validity of the SMSE-PBP (N=349)**

| **Dimensions/Factors** | **Observational variables** | **B** | **β** | ***P*** **Value** | **R-square** | **CR** | **AVE** |
| --- | --- | --- | --- | --- | --- | --- | --- |
| **Dimension 1** |  |  |  |  |  |  |  |
| Factor 1 | Item 1 | 1.00 | 0.32 |  | 0.10 | .76 | .56 |
|  | Item 2 | 1.75 | 0.56 | <.001 | 0.31 |  |  |
|  | Item 3 | 2.15 | 0.66 | <.001 | 0.43 |  |  |
|  | Item 4 | 2.25 | 0.82 | <.001 | 0.67 |  |  |
|  | Item 5 | 1.73 | 0.53 | <.001 | 0.28 |  |  |
| Factor 2 | Item 9 | 1.00 | 0.70 |  | 0.49 | .81 | .69 |
|  | Item 10 | 0.98 | 0.72 | <.001 | 0.51 |  |  |
|  | Item 11 | 0.95 | 0.72 | <.001 | 0.52 |  |  |
| Factor 3 | Item 12 | 1.00 | 0.76 |  | 0.57 | .91 | .87 |
|  | Item 13 | 1.10 | 0.78 | <.001 | 0.61 |  |  |
|  | Item 14 | 0.97 | 0.78 | <.001 | 0.61 |  |  |
|  | Item 15 | 1.02 | 0.75 | <.001 | 0.57 |  |  |
|  | Item 16 | 1.00 | 0.80 | <.001 | 0.65 |  |  |
| **Dimension 2** |  |  |  |  |  |  |  |
| Factor 4 | Item 1 | 1.00 | 0.63 |  | 0.39 | .84 | .74 |
|  | Item 2 | 1.06 | 0.78 | <.001 | 0.60 |  |  |
|  | Item 3 | 1.05 | 0.77 | <.001 | 0.60 |  |  |
|  | Item 6 | 0.96 | 0.70 | <.001 | 0.48 |  |  |
| Factor 5 | Item 12 | 1.00 | 0.65 |  | 0.42 | .92 | .85 |
|  | Item 13 | 0.95 | 0.56 | <.001 | 0.32 |  |  |
|  | Item 14 | 0.98 | 0.63 | <.001 | 0.40 |  |  |
|  | Item 15 | 1.07 | 0.72 | <.001 | 0.53 |  |  |
|  | Item 42 | 1.09 | 0.74 | <.001 | 0.55 |  |  |
|  | Item 43 | 1.09 | 0.79 | <.001 | 0.63 |  |  |
|  | Item 44 | 1.28 | 0.81 | <.001 | 0.66 |  |  |
|  | Item 45 | 1.20 | 0.78 | <.001 | 0.61 |  |  |
| Factor 6 | Item 19 | 1.00 | 0.77 |  | 0.60 | .95 | .93 |
|  | Item 20 | 1.00 | 0.77 | <.001 | 0.60 |  |  |
|  | Item 21 | 0.99 | 0.78 | <.001 | 0.61 |  |  |
|  | Item 22 | 1.10 | 0.83 | <.001 | 0.68 |  |  |
|  | Item 23 | 1.08 | 0.83 | <.001 | 0.68 |  |  |
|  | Item 24 | 1.09 | 0.81 | <.001 | 0.66 |  |  |
|  | Item 25 | 1.06 | 0.83 | <.001 | 0.69 |  |  |
|  | Item 26 | 1.11 | 0.85 | <.001 | 0.71 |  |  |
|  | Item 27 | 1.10 | 0.84 | <.001 | 0.71 |  |  |
|  | Item 28 | 0.94 | 0.76 | <.001 | 0.58 |  |  |
|  | Item 38 | 0.98 | 0.75 | <.001 | 0.56 |  |  |
| Factor 7 | Item 29 | 1.00 | 0.75 |  | 0.56 | .87 | .79 |
|  | Item 30 | 1.06 | 0.71 | <.001 | 0.50 |  |  |
|  | Item 31 | 0.75 | 0.61 | <.001 | 0.38 |  |  |
|  | Item 32 | 1.16 | 0.71 | <.001 | 0.50 |  |  |
|  | Item 33 | 1.16 | 0.86 | <.001 | 0.73 |  |  |
| Factor 8 | Item 34 | 1.00 | 0.70 |  | 0.49 | .87 | .79 |
|  | Item 35 | 1.18 | 0.79 | <.001 | 0.63 |  |  |
|  | Item 36 | 1.15 | 0.80 | <.001 | 0.64 |  |  |
|  | Item 37 | 1.19 | 0.72 | <.001 | 0.52 |  |  |
| **Dimension 3** |  |  |  |  |  |  |  |
| Factor 9 | Item 5 | 1.00 | 0.78 |  | 0.61 | .94 | .94 |
|  | Item 7 | 1.02 | 0.82 | <.001 | 0.68 |  |  |
|  | Item 8 | 1.04 | 0.82 | <.001 | 0.67 |  |  |
|  | Item 9 | 1.07 | 0.76 | <.001 | 0.57 |  |  |
|  | Item 10 | 1.12 | 0.88 | <.001 | 0.77 |  |  |
|  | Item 11 | 1.09 | 0.84 | <.001 | 0.70 |  |  |
|  | Item 12 | 1.17 | 0.88 | <.001 | 0.78 |  |  |
| Factor 10 | Item 14 | 1.00 | 0.72 |  | 0.52 | .95 | .93 |
|  | Item 15 | 1.02 | 0.76 | <.001 | 0.58 |  |  |
|  | Item 16 | 1.05 | 0.83 | <.001 | 0.70 |  |  |
|  | Item 18 | 1.09 | 0.85 | <.001 | 0.73 |  |  |
|  | Item 19 | 1.05 | 0.84 | <.001 | 0.71 |  |  |
|  | Item 20 | 1.01 | 0.83 | <.001 | 0.69 |  |  |
|  | Item 21 | 1.03 | 0.80 | <.001 | 0.64 |  |  |
|  | Item 23 | 1.09 | 0.86 | <.001 | 0.74 |  |  |
| **Dimension 1** |  |  |  |  |  |  |  |
| Factor 1 |  | 1.00 | 0.59 |  | .34 |  |  |
| Factor 2 |  | 3.45 | 0.96 | <.001 | .93 |  |  |
| Factor 3 |  | 3.44 | 0.94 | <.001 | .87 |  |  |
| **Dimension 2** |  |  |  |  |  |  |  |
| Factor 4 |  | 1.00 | 0.80 |  | .64 |  |  |
| Factor 5 |  | 1.07 | 0.96 | <.001 | .92 |  |  |
| Factor 6 |  | 1.06 | 0.68 | <.001 | .46 |  |  |
| Factor 7 |  | 1.13 | 0.88 | <.001 | .77 |  |  |
| Factor 8 |  | 1.09 | 0.93 | <.001 | .87 |  |  |
| **Dimension 3** |  |  |  |  |  |  |  |
| Factor 9 |  | 1.00 | 0.92 |  | .85 |  |  |
| Factor 10 |  | 1.13 | 0.95 | <.001 | .90 |  |  |

Abbreviations: SMSE-PBP, Self-Management Self-Efficacy Scale for Premature Birth Prevention; β, standardized regression coefficient; CR, construct reliability; AVE, average variance extracted.

Dimension 1, Pre-pregnancy SMSE-PBP; Dimension 2, Pregnancy SMSE-PBP; Dimension 3, Hospital SMSE-PBP; F1, proactive lifestyle before pregnancy; F2, proactive problem-specific management before pregnancy; F3, proactive collaboration before pregnancy; F4, proactive lifestyle during pregnancy; F5, proactive collaboration during pregnancy; F6,, reactive management of risk symptom recognition during pregnancy; F7, reactive management according to risk symptoms during pregnancy; F8, self-monitoring of risk symptoms during pregnancy; F9, proactive collaboration and tracking management of symptoms after hospital admission; F10, proactive support and reactive management of the disease after discharge.

^a^*P*<.010
