# Supplementary material for: Development and validation of a self-management self-efficacy scale for premature birth prevention (SMSE-PBP) for women of childbearing age
Source: BMC Womens Health. 2024 Feb 20;24:134. doi: 10.1186/s12905-024-02964-w (PMC10877791; doi:10.1186/s12905-024-02964-w)
Supplement: Supplementary file 2 — Appendix S2. Self-Management Self-Efficacy Scale for Premature Birth Prevention (English Version). [file 12905_2024_2964_MOESM2_ESM.docx]

**Additional file 2**

**Appendix S2. Self-Management Self-Efficacy Scale for Premature Birth Prevention (English Version)**

Please read the following statements while assuming **‘If I am preparing for pregnancy’** and check the boxes that align with your thoughts.

‘Premature birth’ refers to an early birth between 20 and 37 weeks of gestation.

‘Prenatal care’ refers to the health care that pregnant women receive at an obstetrics department or public health center.

1=I can hardly do it, 2=I cannot do it well, 3=I can do it moderately, 4=I can do it well, 5=I can do it very well

| **No** | **If I am preparing for pregnancy,** | **1** | **2** | **3** | **4** | **5** |
| --- | --- | --- | --- | --- | --- | --- |
| **01** | I can adjust to a healthy weight. |  |  |  |  |  |
| **02** | I can regularly consume foods that contain a balanced amount of recommended nutrients. |  |  |  |  |  |
| **03** | I can manage stress to prepare for a healthy pregnancy. |  |  |  |  |  |
| **04** | I can manage my daily life in a healthy way. |  |  |  |  |  |
| **05** | I can avoid exposure to products or environmental hazards that can affect pregnancy. |  |  |  |  |  |
| **06** | If I have chronic diseases such as high blood pressure, diabetes, or anemia, I can self-manage my condition to have a healthy pregnancy. |  |  |  |  |  |
| **07** | If I have a chronic illness, I can consume the necessary diet according to my condition. |  |  |  |  |  |
| **08** | I can consume folic acid supplements (a type of vitamin B required for pregnancy) or foods with high folic acid components. |  |  |  |  |  |
| **09** | I can prevent infectious diseases or receive treatment if infected. |  |  |  |  |  |
| **10** | I can receive pre-pregnancy health management with my partner. |  |  |  |  |  |
| **11** | I can check and receive vaccines for rubella and hepatitis B to confirm immunity (antibodies).  *Rubella is an infectious disease that can cause fetal deformities in case of pregnancy infections. |  |  |  |  |  |
| **12** | If I have mental health problems, I can receive consultation or management from a healthcare professional for pregnancy planning. |  |  |  |  |  |
| **13** | If I have chronic diseases, I can receive consultation or management from a healthcare professional for pregnancy planning. |  |  |  |  |  |

Please read the following statements while assuming **‘If I am pregnant’** and check the boxes that align with your thoughts.

‘Premature birth’ refers to an early birth between 20 and 37 weeks of gestation.

‘Prenatal care’ refers to the health care received by pregnant women at an obstetrics department or public health center.

1=I can hardly do it, 2=I cannot do it well, 3=I can do it moderately, 4=I can do it well, 5=I can do it very well

| No | **If I am pregnant,** | **1** | **2** | **3** | **4** | **5** |
| --- | --- | --- | --- | --- | --- | --- |
| 14 | I can manage stress for a healthy pregnancy. |  |  |  |  |  |
| 15 | I can manage daily life in a healthy way for pregnant women. |  |  |  |  |  |
| 16 | I can take preventive measures to prevent urinary or reproductive tract infections such as cystitis or vaginitis. |  |  |  |  |  |
| 17 | I can regularly consume foods that contain a balanced amount of recommended nutrients for pregnant women. |  |  |  |  |  |
| 18 | I can take preventive measures to avoid epidemics. |  |  |  |  |  |
| 19 | I can receive flu vaccination. |  |  |  |  |  |
| 20 | I can avoid risk factors (abdominal shock, severe stress, smoking, drug use, etc.) to prevent the detachment of the placenta from to the uterus. |  |  |  |  |  |
| 21 | If the cervical length of the uterus is shortened according to test results in the middle of pregnancy, I can receive the recommended treatment. |  |  |  |  |  |
| 22 | If I have mental health problems, I can receive consultation or management from a healthcare professional. |  |  |  |  |  |
| 23 | If there are any physical health problems, I can receive consultation and management from a healthcare professional. |  |  |  |  |  |
| 24 | If I become pregnant over the age of 35, I can visit a women’s hospital or public health center more frequently for prenatal management. |  |  |  |  |  |
| 25 | I can ask my family or acquaintances for help to manage a healthy pregnancy. |  |  |  |  |  |
| 26 | I can early detect regular or frequent lower abdominal pain or tightening as signs of premature delivery |  |  |  |  |  |
| 27 | I can early detect water-like fluid (amniotic fluid) coming out of the vagina, unlike usual, as signs of premature delivery. |  |  |  |  |  |
| 28 | I can early detect mucous mixed with blood coming out of the vagina as signs of premature delivery. |  |  |  |  |  |
| 29 | I can early detect high blood pressure as a warning sign of premature delivery. |  |  |  |  |  |
| 30 | I can early detect severe symptoms of pregnancy-induced hypertension (severe headache, blurred vision) as warning signs of premature delivery. |  |  |  |  |  |
| 31 | I can early detect severe fatigue as a sign of premature delivery. |  |  |  |  |  |
| 32 | I can early detect a low or absent number of fetal movements (fetal kicks) as a sign of premature delivery. |  |  |  |  |  |
| 33 | I can early detect urinary pain or burning during urination as a sign of premature delivery. |  |  |  |  |  |
| 34 | I can early detect low or high blood sugar levels as a sign of premature delivery. |  |  |  |  |  |
| 35 | I can early detect shortness of breath as a sign of premature delivery. |  |  |  |  |  |
| 36 | I can observe and determine whether lower abdominal pain or tightening is labor contractions. |  |  |  |  |  |
| 37 | If the risk symptoms of premature birth are detected, I can quickly call or visit a hospital. |  |  |  |  |  |
| 38 | If the risk symptoms of premature birth are detected, I can take immediate rest. |  |  |  |  |  |
| 39 | If the risk symptoms of premature birth are detected, I can avoid sexual activity. |  |  |  |  |  |
| 40 | If the risk symptoms of premature birth are detected, I can know and choose a tertiary medical institution (such as a university hospital) capable of managing premature infants. |  |  |  |  |  |
| 41 | If the risk symptoms of premature birth are detected, I can visit the hospital frequently to receive early diagnosis, testing, and treatment. |  |  |  |  |  |
| 42 | I can monitor recommended weight gain based on body mass index by periodically measuring my weight. |  |  |  |  |  |
| 43 | If I have high blood pressure, I can monitor normal and abnormal blood pressure levels by periodically measuring my blood pressure. |  |  |  |  |  |
| 44 | If I have diabetes, I can monitor normal and abnormal blood sugar levels by periodically measuring my blood sugar. |  |  |  |  |  |
| 45 | From 28 weeks of pregnancy, I can observe normal and abnormal fetal movements by conducting daily fetal movement tests. |  |  |  |  |  |

Please read the following statements while assuming **‘If I am hospitalized during pregnancy’** and check the boxes that align with your thoughts.

‘Premature birth’ refers to an early birth between 20 and 37 weeks of gestation.

‘Prenatal care’ refers to the health care that pregnant women receive at an obstetrics department or public health center.

1=I can hardly do it, 2=I cannot do it well, 3=I can do it moderately, 4=I can do it well, 5=I can do it very well

| No | **If I am hospitalized during pregnancy,** | **1** | **2** | **3** | **4** | **5** |
| --- | --- | --- | --- | --- | --- | --- |
| 46 | Depending on the risk of premature birth, I can manage personal hygiene activities. |  |  |  |  |  |
| 47 | I can follow diagnosis tests according to my medical condition. |  |  |  |  |  |
| 48 | To monitor the periodic health status of the fetus and pregnant woman, I can follow tests as required. |  |  |  |  |  |
| 49 | I can periodically self-check for warning signs and symptoms. |  |  |  |  |  |
| 50 | I can participate and adhere to treatment goals and plans according to my medical condition. |  |  |  |  |  |
| 51 | I can participate in the decision-making process for determining the timing of childbirth. |  |  |  |  |  |
| 52 | I can faithfully adhere to the treatment plan set together. |  |  |  |  |  |
| 53 | I can acquire information on self-management for preventing premature birth. |  |  |  |  |  |
| 54 | I can request support from family or acquaintances. |  |  |  |  |  |
| 55 | I can request necessary support from healthcare professionals. |  |  |  |  |  |
| 56 | After discharge, I can agree to frequent prenatal care and adhere to it. |  |  |  |  |  |
| 57 | After discharge, I can acquire information on the warning signs of premature labor and self-management techniques at home. |  |  |  |  |  |
| 58 | After discharge, I can faithfully adhere to the treatment plan at home. |  |  |  |  |  |
| 59 | After discharge, I can adjust my daily activities to prevent the recurrence of premature birth symptoms. |  |  |  |  |  |
| 60 | After discharge, I can quickly call or visit the hospital if signs of preterm labor are detected. |  |  |  |  |  |

*The English translation has not been validated for use in English-speaking settings.

Note:

Pre-pregnancy SMSE-PBP: Proactive lifestyle before pregnancy, items 1 to 5; proactive problem-specific management before pregnancy, items 6 to 8; and proactive collaboration before pregnancy, items 9 to 13.

Pregnancy SMSE-PBP: Proactive lifestyle during pregnancy, items 14 to 17; proactive collaboration during pregnancy, items 18 to 25; reactive management of risk symptom recognition during pregnancy, items 26 to 36; reactive management according to risk symptoms during pregnancy, items 37 to 41; and self-monitoring of risk symptoms during pregnancy, items 42 to 45.

Hospital SMSE-PBP: proactive collaboration and tracking management of symptoms after hospital admission, items 46 to 52, and proactive support and reactive management of the disease after discharge, items 53 to 60.
